# Atypical Obstructive Pseudotumors in AIDS as the Initial Manifestation of Gastrointestinal Histoplasmosis

**DOI:** 10.7759/cureus.7775

**Published:** 2020-04-22

**Authors:** Alejandro Hallo, Malena Camacho, Alejandra Rojas, Andres Mayancela, Alberto Castillo

**Affiliations:** 1 Internal Medicine, Hospital de Especialidad Eugenio Espejo, Quito, ECU; 2 Internal Medicine, Hospital de Especialidades Eugenio Espejo, Quito, ECU; 3 Investigacion, Universidad Central del Ecuador, Quito, ECU; 4 Internal Medicine, Hospital Eugenio Espejo, Quito, ECU

**Keywords:** histoplasmosis, opportunistic mycoses, aids

## Abstract

Histoplasma capsulatum is a dysmorphic fungus distributed worldwide commonly associated with pulmonary histoplasmosis. We report the case of an unusual presentation of gastrointestinal histoplasmosis leading to the obstruction of the intestinal lumen in a 30-year-old female, HIV positive, admitted to the hospital due to chronic abdominal pain and constipation. An initial abdominal CT revealed a mass in the sigmoid colon. A further colonoscopy showed an infiltrating, friable mass obstructing 80% of the lumen staining positive for H. capsulatum. The unspecific nature of the patient's symptoms along with the unusual presentation of the infection raises awareness about the importance of including new pathologies to differential diagnoses when treating AIDS patients.

## Introduction

*Histoplasma capsulatum* is one of the most common opportunistic pathogens affecting AIDS patients with a CD4 <100 [1]. Histoplasmosis results from impaired infection control from macrophages and CD4 [2]. Throughout the years, the use of antiretroviral therapy (ART) has modified the symptoms, location, and evolution of this disease as well as many other infections. Moreover, many AIDS-related infectious diagnoses occurred incidentally due to their often unspecific symptoms [3]. 

We report a case of a patient with a CD4 count of 64 cells/mL with chronic constipation from gastrointestinal histoplasmosis causing a pseudotumor obstructing 80% of the intestinal lumen.

## Case presentation

A 30-year-old cachectic woman with a past medical history of HIV and poor compliance with ART came to the ED due to diffuse abdominal pain and low-grade fever.

Physical examination revealed diffuse abdominal pain without rebound tenderness, jaundice, or palpable liver. The patient reported chronic constipation and weight loss (12 kg) over one year before the admission and denied respiratory symptoms.

Initial laboratory showed microcytic anemia (Hb 6.5, Hct 20%, MCV 67 fL), CD4 count: 64 cells, viral load: 20 copies/mL, total proteins: 4.28, albumin: 1.21. Fecal occult blood was positive and no ova or parasites were seen in the stool sample.

An abdominal CT showed thickening of the intestinal wall and mesenteric lymphadenopathy.

**Figure 1:**
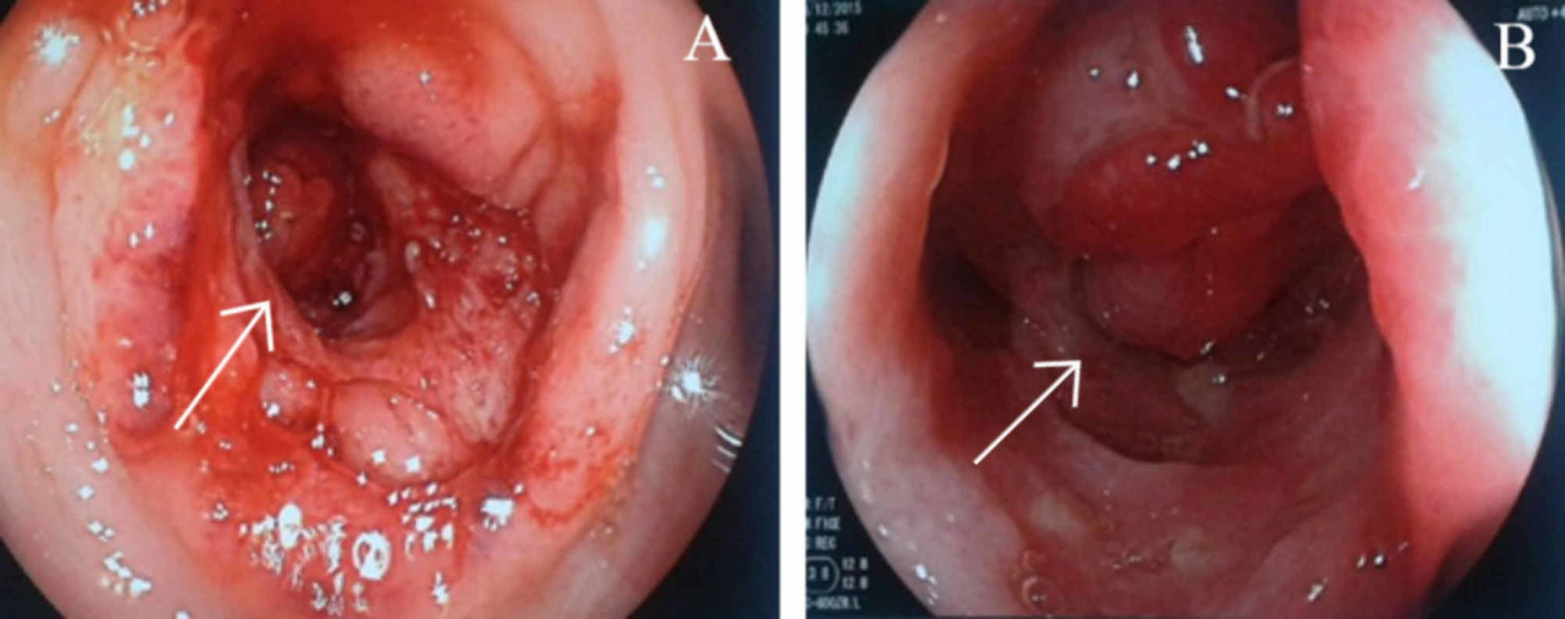
Sigmoid colon biopsy. Ulcerated, irregular, friable, deep lesion with congestive edges, causing obstruction of 80% of the intestinal lumen.

An upper digestive endoscopy was performed due to the patient’s CT results showing erythematous gastric mucosa in the antrum. Later, a colonoscopy revealed a friable pseudo tumoral mass in the sigmoid colon obstructing 80% of the lumen (Figure 1).

Empirical treatment for tuberculosis (TB) was started as the patient came from an endemic area.

**Figure 2:**
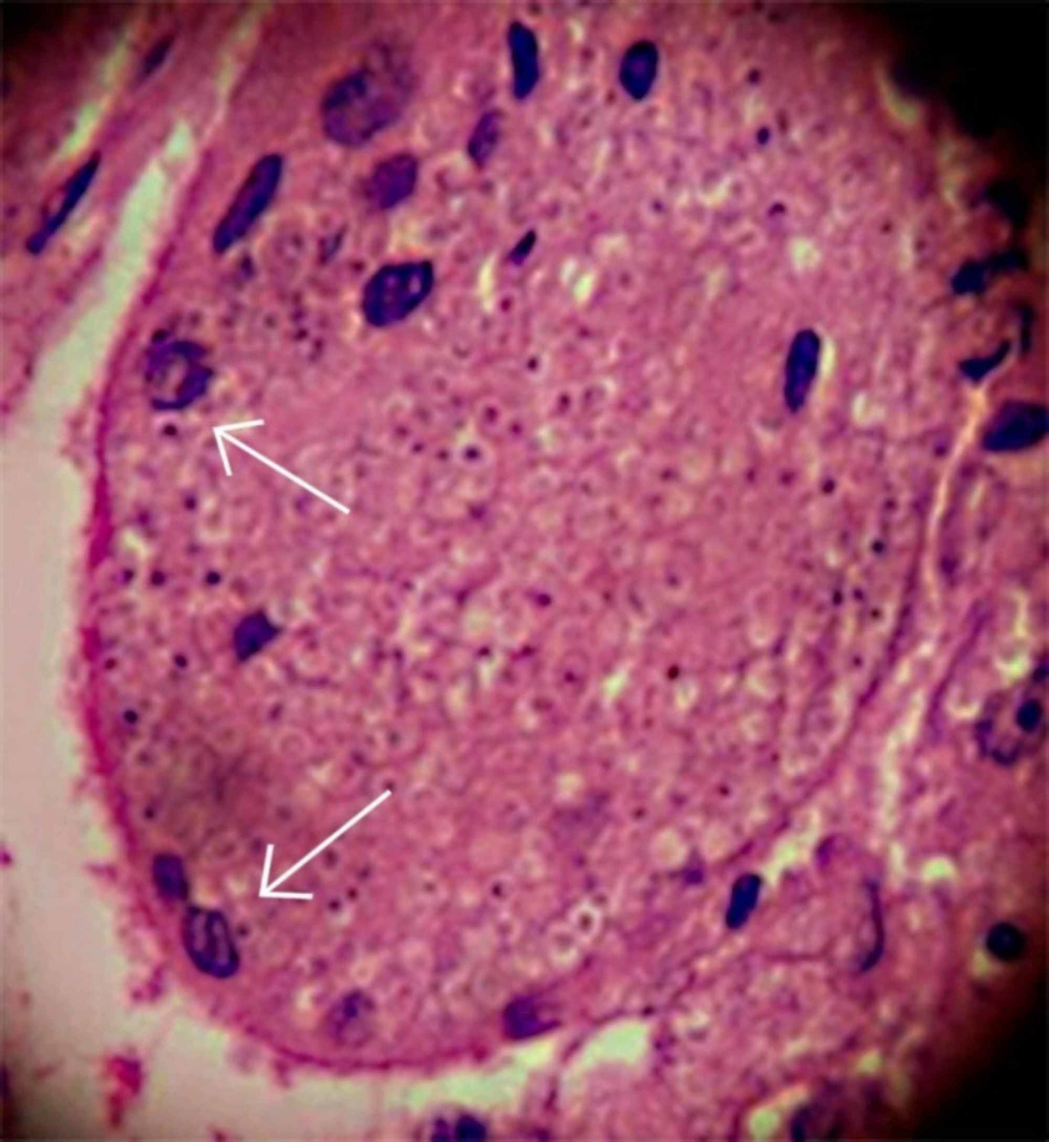
Histopathological biopsy study obtained from pseudotumoral mass in the sigmoid colon. *Histoplasma capsulatum* highlighted with periodic acid-Schiff (PAS) stain (arrow).

The histopathological examination of the pseudotumoral mass identified *H. capsulatum* (Figure 2). Therefore, the anti-TB treatment was replaced by amphotericin B 25 mg daily for 25 days.

Amphotericin B was stopped after 14 days due to the improvement in the patient’s symptoms and then switched to itraconazole 200 mg every eight hours for four days, followed by 200 mg twice daily for one year with the successful regression of the symptoms.

## Discussion

This case report is unique because the infection presented as a pseudotumoral mass obstructing 80% of the colonic lumen. Although we identified six cases of intestinal *H. capsulatum* in immunosuppressed patients reported in the literature, all of those cases presented as intestinal ulcers rather than obstructive entities [2-7].

Histoplasmosis is a common comorbidity in AIDS patients [8]; however, this condition can also be reported in immunocompetent patients [4, 9].

The infection is caused by breathing spores from soil contaminated by bird and bat droppings [1, 4, 9]. For this reason, pulmonary histoplasmosis is the classical presentation of the infection. Other organs like the skin and adrenal glands can also be affected. The gastrointestinal system can be affected in the disseminated form of the disease [1]. 

In our patient, the diagnosis of intestinal histoplasmosis was incidental because there was no preceding pulmonary presentation which usually leads to a suspicion of a disseminated condition.

Some cases in the literature report diarrhea and abdominal pain as the most common symptoms [2-7]; however, our patient presented with chronic constipation. This atypical symptom may be the result of the mechanical obstruction of the lumen by the mass.

The detection of urine *H. capsulatum* antigens is used in the diagnosis of the pulmonary infection and the disseminated presentation [1, 8, 10].

Intestinal ulcers are common colonoscopic findings in gastrointestinal histoplasmosis [2, 4-7]. An obstructive lesion was reported only by Winn et al. [4].

## Conclusions

We presented a rare case of intestinal histoplasmosis by *H. capsulatum* presented in the form of a pseudotumoral obstructive mass. This report raises awareness to consider this pathology as a new differential diagnosis in patients with AIDS due to the increase in atypical presentations of infections.
